# Prevalence and associated risk factors assessment of bovine fasciolosis in the Imbo Region, Burundi

**DOI:** 10.1007/s00436-023-08040-w

**Published:** 2023-12-13

**Authors:** Sylvère Nkurunziza, Gérard Nishemezwe, Jean-Bosco Ntirandekura, Pascal Niyokwizera, Lionel Nyabongo, Amos Omore, Rose Odhiambo

**Affiliations:** 1 Department of Parasitology, National Veterinary Laboratory, P.O. Box 227, Bujumbura, Burundi; 2 Department of Biological Sciences, Faculty of Sciences, Egerton University, Njoro, Nakuru, Kenya; 3 Department of Animal Health and Productions, Faculty of Agronomy and Bio-Engineering, University of Burundi, Bujumbura, Burundi; 4 National Veterinary Laboratory, Bujumbura, Burundi; 5 International Livestock Research Institute, ILRI Burundi Office, Bujumbura, Burundi; 6 International Livestock Research Institute, ILRI Tanzania Office, Dar Es Salaam, Tanzania; 7 Global Health and Gender, Immunology and Parasitology, Egerton University, Njoro, Nakuru, Kenya

**Keywords:** Fasciolosis, Cattle, Prevalence, Risk factors, Burundi

## Abstract

Fasciolosis is a zoonosis that limits the productivity of ruminants worldwide, but there is a lack of information on its occurrence in Burundi. Therefore, this study aimed to fill the information gap by determining the prevalence and risk factors associated with bovine fasciolosis in the Imbo Region of Burundi. Two prevalence studies were conducted in parallel in the five communes of the five provinces in the Imbo region. In the first study, a total of 426 fecal samples were collected from randomly selected cattle farms and microscopically examined to determine *Fasciola* egg burden. Survey data on cattle husbandry were collected from owners of these cattle and analyzed to determine the risk factors for bovine fasciolosis. In the second study, 467 cattle were randomly selected in abattoirs and their livers were examined postmortem to determine liver fluke burdens. Data were entered separately into Microsoft Excel and analyzed using R software. The overall prevalence of bovine fasciolosis was 47.7% (42.9–52.4, 95% CI) for microscopic examination and 33.2% (28.9–37.5, 95% CI) for postmortem examinations. The majority of positive cattle (60.6%) had light intensity infections as determined by eggs per gram of feces (epg). Postmortem examinations corroborated these results and indicated that 80% of cattle had light intensity infections. Chi-square analysis showed a statistical association with the presence of bovine fasciolosis and the age, sex, and origin of cattle and the practices of cattle owners (*P* < 0.05).

## Introduction

Fasciolosis is a helminth infection of cattle, buffaloes, sheep, goats, horses, and human of all ages caused by ingestion of encysted metacercaria of liver flukes from the genus *Fasciola* (Ardo et al. 2014; Figtree et al. 2015; Najib et al. 2020). Livestock acquire infection through ingestion of metacercaria that are attached to forage or by drinking water contaminated by metacercaria attached to soil particles or vegetative debris (Irsik et al. 2008). Humans get infected not directly from livestock, but through ingestion of encysted metacercaria that are attached on raw vegetables or certain aquatic plants. The infection may also be acquired through consumption of water contaminated by encysted metacercaria, or food items washed with such water (Ibrahim 2017) or consumption of contaminated water of human sewage (Aleixo et al. 2015). According to the World Health Organization (WHO), human fasciolosis is classified as a Neglected Tropical Disease (NTD) (Ai et al. 2017; Abah et al. 2019). Human fasciolosis is a major public health problem in several areas of the world, with human cases being increasingly reported from Europe, the Americas, Oceania, India and Africa (Aksoy et al. 2005; Soliman 2008). In countries such as Bolivia, Peru and Egypt, human fasciolosis is considered hyper-endemic and its prevalence is estimated to be greater than 70% through the analysis of stool samples and 100% through serological means (Aleixo et al. 2015). In Bolivia and Egypt, children are mainly infected and get contaminated by eating edible plants while taking care of the family herd (Mas-Coma 2014; Aleixo et al. 2015). Human and animal fasciolosis can have serious pathological consequences in the liver, with severe damage due to migration of the flukes through the liver or other organs (Najib et al. 2020). Clinically, fasciolosis infection in animals and humans is known to cause bile duct inflammation and biliary obstruction *(*Sah et al. 2017). Fasciolosis is endemic worldwide, including Europe (Isabirye et al. 2012), the Americas (Kleiman et al. 2007), Asia (Nguyen et al. 2011), Middle East, Oceania (Isabirye et al. 2012) and Africa (Abunna et al. 2010). The geographical distribution of fasciolosis depends on the distribution of suitable aquatic species of snails *Lymnaea truncatula* and *Lymnaea natalensis* which serve as an intermediate host (Boray and Love 2007; Ibrahim 2017)*.* In sub-Saharan Africa (SSA), fasciolosis has been reported in East Africa (Dida et al. 2014), West Africa (Elelu et al. 2016), South Africa (Nyirenda et al. 2019), and North Africa (Elshraway and Mahmoud 2017). Fasciolosis is known to be prevalent and cause serious problem in sheep, goats and cattle (Soliman 2008). The economic losses of fasciolosis infection in animals include mortality, morbidity, the reduction of growth rate, the condemnations of liver, the increasing susceptibility to secondary infections, the cost of implementing control or treatment protocols, a reduction of productivity and a decrease of fertility and milk production (Bernardo et al. 2011). In the East Africa region, studies conducted in ruminants showed a fasciolosis prevalence of 9–31% in Tanzania (Mellau et al. 2010) and 7–26% in Kenya (Mungube et al. 2006). Studies conducted on the epidemiology of fasciolosis among animals have shown that the risk factors, including age, sex, breed and livestock management have a significant influence on the prevalence of fasciolosis (Phiri et al. 2005; Keyyu et al. 2006; Abunna et al. 2010). In Burundi in general, and specifically in the Imbo region, there is a lack of information on the epidemiology and control of fasciolosis. Reports from veterinary services of Burundi show cases of morbidity in cattle and in small ruminants, and liver fluke burden during abattoir meat inspections (Report from General Directorate of livestock in Burundi 2021). The prevalence of 12.3% of bovine fasciolosis was reported by the veterinary services of Burundi (Report from General Directorate of livestock in Burundi 2021). Therefore, this study was undertaken to establish the prevalence and risk factors associated with bovine fasciolosis in the Imbo Region of Burundi.

## Materials and methods

### Description of study areas

**Fig. 1:**
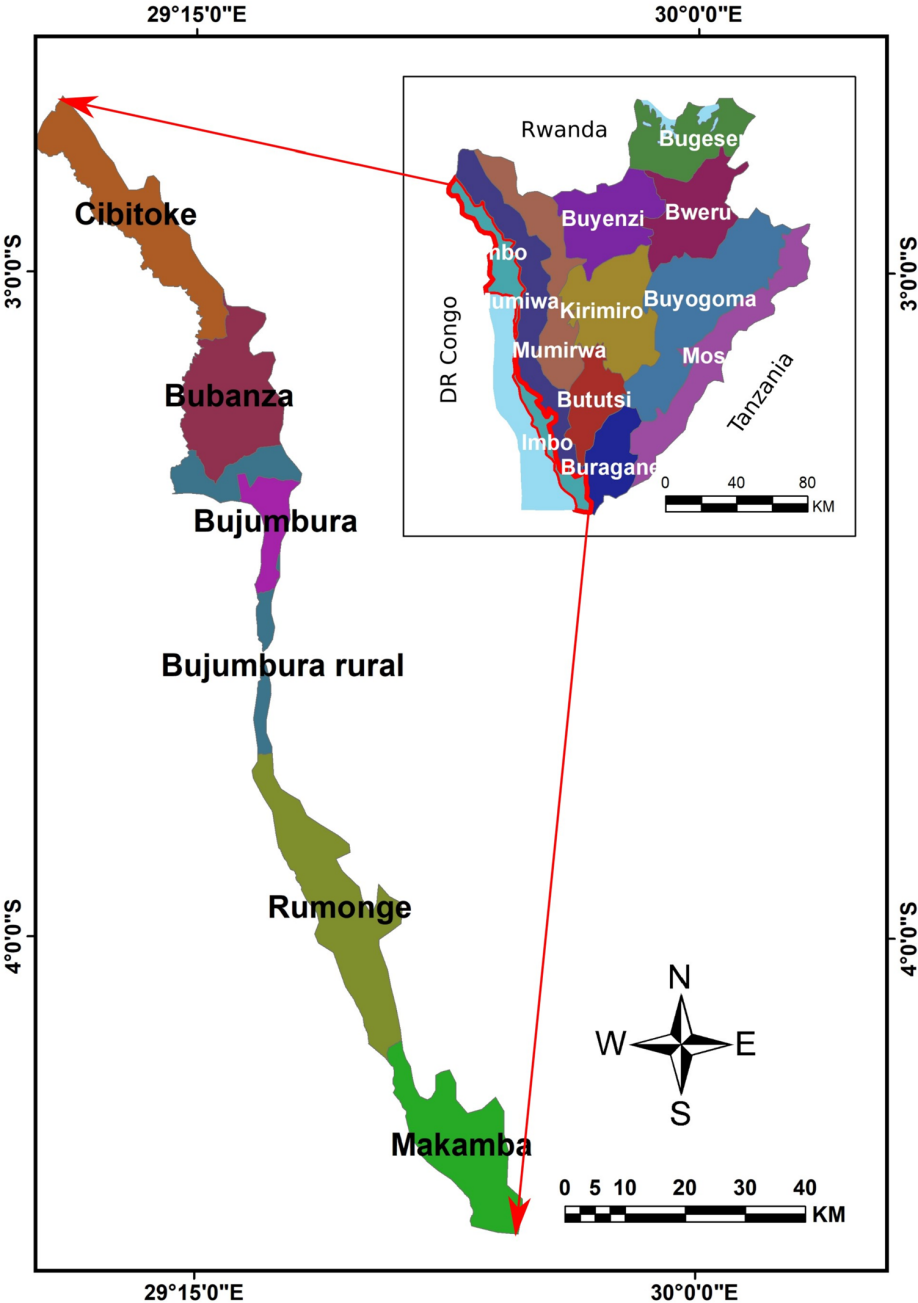
Map of naturals region of Burundi, Imbo Region and its six provinces

The present study was conducted in 5 communes namely Nyanza-lac, Rumonge, Rugombo, Rugazi and Mutimbuzi in 5 provinces respectively namely Makamba, Rumonge, Cibitoke, Bubanza and Bujumbura Rural in the Imbo region of Burundi. The Imbo region extends unequally over 6 provinces composed of 11 rural communes and 3 urban communes of Bujumbura town. The Imbo region is located between 2°48′30″ and 4°20′43″ latitude South and 29°36′3″ longitude East. The Imbo region is the westernmost region and the lowest in altitude of Burundi and extends north from Lake Tanganyika towards the Democratic of Congo (Fig. 1). Imbo region is characterized by a rainfall of 800 to 1100 mm of rain spread over 7 to 8 months such as January, February, March, April, May, October, November and December, but certain parts, especially the north, are chronically arid. The average annual temperature is 25° C (range 15^–^30° C). The relative humidity is estimated at 70%. The Imbo Region is one of the most densely populated of the country (300 inhabitants/km^2^), and its economy is largely dependent on agriculture and livestock (MEEATU 2013).

### Study design and sample size

Two cross-sectional studies were conducted parallelly in the Imbo Region of Burundi from September to December 2022. Firstly, prevalence of bovine fasciolosis was determined using a microscopic technique based on the presence or absence of *Fasciola* eggs stage in the fecal samples collected in the farms. During fecal samples collection, animal level information including sex, breed, body condition scores, origin (communes and provinces) and survey data on cattle husbandry including source of fodder, source of water, feeding system and anthelmintics commonly used to treat bovine fasciolosis were collected from cattle owners to determine the risk factors associated with bovine fasciolosis. The sampled cattle were randomly selected in the households located in 20 hills of 5 communes in 5 provinces of the Imbo region in Burundi. The farmers surveyed were the cattle owners or household heads. In their absence, another adult member of the family, minimum 18 years old, was interviewed. For every cattle owner, one or more cattle heads were sampled depending on the herd size and the inclusion criteria, such as clinical signs including diarrhea, dullness, rough body hair, emaciation, bottle jaw and weight loss. The second study was conducted in the abattoirs to determine the prevalence of bovine fasciolosis based on the presence or absence of the *Fasciola* adult flukes in the bile ducts of the liver of slaughtered cattle using visualization, palpation, and incision of the liver with a knife. The slaughtered cattle were randomly selected in 5 abattoirs of 5 communes in 5 provinces of the Imbo region in Burundi. Each abattoir was chosen in one commune of one province of the Imbo region and these communes and provinces were the same where fecal samples were collected from. The selected cattle for the study were these came from in province where abattoir is located. A multistage random sampling strategy was used for selecting cattle and owners for both studies. Before beginning, the local veterinary services were consulted to obtain a list of provinces, communes, and hills of the Imbo region. Local veterinary services of the hills, communes and provinces selected were consulted to obtain a list of cattle owners on each hill and abattoir on each commune. Based on the lists, the names of provinces, communes, hills, cattle owners, and abattoirs visited were randomly generated using the RAND function in Microsoft Excel. Since there was no study in Burundi to establish the prevalence of bovine fasciolosis, the sample size was determined by taking the prevalence of 50%, absolute precision of 5%, and confidence level of 95% using the formula as reported by Abah et al.(2019): *n* = Required sample size, *t* = confidence level of 95% (Standard value of 1.96), P = Estimated prevalence = 50% (0.5), M = Margin of error at 5% (Standard value of 0.05).

Sample size of cattle from farms for coprological studies:

$$\mathrm{n}=\frac{{t}^{2}*P\left(1-P\right)}{{M}^{2}}=\frac{{1.96}^{2}*0.5\left(1-0.5\right)}{{0.05}^{2}}=384$$

A total of 384 cattle was estimated, but 426 cattle were sampled to maximize the precision.

Sample size of cattle from abattoir for postmortem liver inspection:

$$\mathrm{n}=\frac{{t}^{2}*P\left(1-P\right)}{{M}^{2}}=\frac{{1.96}^{2}*0.5\left(1-0.5\right)}{{0.05}^{2}}=384$$

A total of 384 cattle was estimated, but 467 was sampled to maximize the precision of prevalence.

### Coprological examination and assessment risk factors associated with bovine fasciolosis

Fecal samples were taken directly from the rectum of each cattle of 426 cattle using disposable gloves and placed in a clean screw-cap universal sample bottle, and clearly labeled with number code identification of animal. Date, code number, age, sex, breed, body condition score, origin, clinical signs of each animal sampled and survey data from cattle owners were recorded using web-based mobile phone application, Kobocollect (Version May 20, 2018 Copyright Act of 1998, Sect. 512 of the U.S. Copyright Act). Fecal samples preserved in 10% formalin were transported to the National Veterinary Laboratory of Bujumbura, and stored in a refrigerator at 4 °C (Mequaninit 2021). In the laboratory, a sedimentation technique was used to detect the presence or absence of liver fluke eggs in the fecal samples under an optical microscope (Tulu and Gojam 2018). Five grams of fecal samples was added into 50 ml of water into container 1. The contents were then mixed using a tongue blade and filtered through a tea strainer to remove any large debris into container 2. The filtrate was left to sediment for 5 min. The supernatant was then very carefully discarded. The sediment was then resuspended in 40 ml of water and was left to sediment for 5 min. The supernatant was again very carefully discarded. The sediment was then stained with two drops of methylene blue solution to differentiate the eggs of *Fasciola* species and *Paramphistomum* species or other trematodes where the eggs of *Fasciola* species show a yellowish color while the eggs of *Paramphistomum* species stain blue methylene. The sediment was placed on a slide with a coverslip and viewed under the microscope. Eggs of *Fasciola* species were identified by their morphologic characteristics (operculated with thin shell, broadly ellipsoidal with the estimated measurements of 130–150 Mm in length and 63–90 Mm in breadth, and yellow color) (Hussein et al. 2010; Miller 1997 ; Roepstortf and Nansen 1998). The MacMaster counting technique was used to determine the egg burden of *Fasciola* species as indicated by Miller (1997). Five grams of fecal samples was added into container 1 of 56 ml of flotation liquid (Zinc sulfate) and mixed using a tongue blade. The solution was then passed through a sieve (mesh size 210 mm) into container 2. While swirling the filtrate in container 2, subsample was taken with a Pasteur pipette. Both sides of the Macmaster counting chamber were filled with the subsample. The counting chamber was let to sit for 5 min. The counting chamber was examined under a microscope and all the eggs in the etched area were counted. The number of eggs per gram of feces (epg) was calculated as follows, the number of eggs from both chambers was added and the total was multiplied by 50. The level of infection intensity was classified according to the number of eggs per gram of feces (epg), such as: light infection (epg from 1 to 600), moderate infection (epg between 600 and 1000 eggs), and heavy infection (epg > 1000 eggs) (Tulu and Gojam 2018).

### Abattoir study

At each abattoir, before they are slaughtered, the cattle were selected based on whether they came from the province that this slaughterhouse is located. The visit to each abattoir took a period of 10 days (2 weeks) from Monday to Friday per week at the time from 6:00 to 9:00 AM (3 h per day). A total of 467 cattle were randomly selected during ante-mortem and individual information including date, cattle owner, origin, sex, age, breed, body condition score and clinical signs were recorded in notebooks. During post-mortem inspection, the liver and associated bile ducts and gallbladders were carefully examined for the presence or absence of liver fluke using visualization, palpation and incision with a knife (Kusumarini et al. 2020). Liver lesions were then grouped based on the liver fluke burden into mild infection (1–29 flukes), moderate infection (30–50 flukes) and heavy infection (> 50 flukes) (Mpisana et al. 2022).

### Data analysis

The recorded data from coprological examination, survey and abattoir were entered separately into the Microsoft Excel database and carefully cleaned. The cleaned data was imported into R statistical software for analysis where prevalence, mean, and standard deviation were generated. The chi-square and odds ratio were also computed to assess the association between bovine fasciolosis and sex, age, body condition score, breed, and origin. A 95% confidence interval and *P*-value of less than 0.05 (at the 5% level of significance) were considered to be significant in all analyses (Opio et al. 2021).

## Results

### Prevalence of bovine fasciolosis in two studies

Of the 426 fecal samples examined from cattle, the current study showed 203 cattle (47.7%, CI: 42.9–52.4) positive for fasciolosis in farms from the study region (Table 1). Of the 467 livers from the slaughtered cattle in the study area, 155 livers (33.2%, CI: 28.9–37.5) were found to have liver flukes (Table 1).

**Table 1:** Overall prevalence of bovine fasciolosis in farms (fecal analysis) and abattoirs (liver necropsy) in the study area, Imbo region, Burundi

In farm (fecal analysis)			In abattoir (liver necropsy)		
Cattle examined	Positive cattle	Overall prevalence % (95% CI)	Cattle examined	Positive cattle	Overall prevalence % (95% CI)
426	203	47.7 (42.9–52.4)	467	155	33.2 (28.9–37.5)

*95% CI*, 95% confidence interval

Regarding the level of infection based on eggs per gram of feces (epg), this study showed 123 cattle (60.6%) with light infection, 57 cattle (28.1%) with moderate infection and 23 cattle (11.3%) with heavy infection (Table 2). Regarding level of infection based on liver fluke burden, the study showed 124 cattle (80%) with mild infection, 12 cattle (7.7%) with moderate infection and 19 cattle (12.3%) with heavy infection (Table 3).

**Table 2:** Prevalence of *Fasciola* spp. infection intensity based on eggs per gram of feces (epg) in bovines from the study area (Imbo Region, Burundi)

Severity	Positive cattle	Intensity class	Prevalence % (95% CI)	Mean epg	Std. Dev. of epg	Minimum	Maximum
Light infection	203	123	60.6 (53.9–67.3)	561.3	377.9	100	1600
Moderate infection	203	57	28.1 (21.9–34.3)				
Heavy infection	203	23	11.3 (6.9–15.7)				

*95% CI*, 95% confidence interval, *epg*, eggs per gram of feces, *Std. Dev*., standard deviation

**Table 3:** Prevalence of *Fasciola* spp. infection intensity based on number of liver flukes in the liver if slaughtered cattle from abattoirs of the study area (Imbo Region, Burundi)

Severity	Positive cattle	Intensity class	Prevalence % (95% CI)	Mean liver fluke	Std. Dev. liver fluke	Minimum	Maximum
Mild infection	155	124	80 (73.7–86.3)	23.2	35.9	1	210
Moderate infection	155	12	7.7 (3.5–12)				
Heavy infection	155	19	12.3 (7.1–17.4)				

*95% CI*, 95% confidence interval, *Std. Dev*., standard deviation

### Risk factors that influence fasciolosis in cattle

Based on the origin of the fecal samples from the Imbo region, the current study showed that 38 cattle (30.7%) in Rugombo commune of Cibitoke province, 36 cattle (40.9%) in Rugazi commune of Bubanza province, 42 cattle (50.6%) in Nyanza-Lac Commune of Makamba province, 32 cattle (56.1%) in Rumonge commune of Rumonge province and 55 cattle (74.3%) in Mutimbuzi commune of Bujumbura rural province were positive for fasciolosis. This study reported a statistical difference (*P*-value < 0.05) between the prevalence of fasciolosis according to the origin of the fecal sample (χ2 = 39 and *P*-value = 6.9e-09 means < 0.01) (Table 4).

**Table 4:** Prevalence of bovine fasciolosis based on fecal samples origin in Imbo region, Burundi

Origin of fecal sample	Number of cattle tested	− ve	+ ve	prevalence % (95% CI)	χ2	df	*P*-value
					39	4	6.9e-09(< 0.01)*
Rugombo	124	86	38	30.7 (22.5–38.8)			
Nyanza-lac	83	41	42	50.6 (39.9–61.4)			
Rumonge	57	25	32	56.1 (43.3–69)			
Rugazi	88	52	36	40.9 (30.6–51.2)			
Mutimbuzi	74	19	55	74.3 (64.4–84.3)			

* − ve*, negative cattle; + *ve*, positive cattle; *95% CI*, 95% confidence interval; *χ*^*2*^, chi-square; *df*, degree of freedom; *: significant difference at *p* < 0.05

Based on abattoir location, this study showed the prevalence of 53.2% in Rubirizi abattoir of Mutimbuzi commune in Bujumbura Rural Province, 51.2% in Rwaba abattoir of Nyanza-Lac commune in Makamba province, 31.7% in Tr4 abattoir of Rugombo commune in Cibitoke province, 16.8% in Muzinda abattoir of Rugazi commune in Bubanza province and 11.5% in Birimba abattoir of Rumonge commune in Rumonge province. Therefore, in the univariate analysis using chi-square test, there was a statistically significant (*P*-value < 0.05) difference between abattoir location (χ2 = 67.6, *P*-value = 7.2e-16 means < 0.01) and the prevalence of *Fasciola* spp. fasciolosis in cattle (Table 5).

**Table 5:** Prevalence of bovine fasciolosis based on abattoir location

Abattoir location	Livers examined	− ve	+ ve	prevalence % (95% CI)	χ2	df	*P*-value
					67.6	4	7.2e-16 (< 0.01)*
Rwaba abattoir in Nyanza-Lac	43	21	22	51.2 (36.2–66.1)			
Birimba abattoir in Rumonge	87	77	10	11.5 (4.8–18.2)			
Tr4 abattoir in Rugombo	60	41	19	31.7 (19.9–43.4)			
Muzinda abattoir in Rugazi	119	99	20	16.8 (10.1–23.5)			
Rubirizi abattoir in Mutimbuzi	158	74	84	53.2 (45.4–60.9)			

* − ve*, negative cattle; + *ve*, positive cattle; *95% CI*, 95% confidence interval; *χ2*, chi-square, *df*, degree of freedom; *: significant difference at *p* < 0.05

This study showed that the prevalence of bovine fasciolosis based on sex and age was higher for female (51.6%) than male (28.8%); and was higher for adult 2–4 years (58.1%) than old > 4 years (42.7%) and young < 2 years (33.9%) cattle. The univariate analysis showed that risk factors such as sex (χ2 = 12.6, *P*-value = 0.3e-3 means < 0.01), and age (χ2 = 19.7, *P*-value = 5.1e-07 means < 0.01) were statistically associated with the bovine prevalence of fasciolosis in this study. Furthermore, female (OR = 2.6; IC: 1.5–4.6) and adult cattle 2–4 years (OR = 2.7; IC: 1.7–4.3) were likely to be more at risk to get bovine fasciolosis in the study area (Table 6). In a multivariate logistic regression analysis, this study showed that the risk factors such as sex (*P*-value = 0.01) and age (*P*-value = 0.02) were statistically associated with the prevalence of bovine fasciolosis (Table 7).

**Table 6:** Univariate analysis of risk factors associated with bovine fasciolosis

Risks factors	Cattle tested	− ve	+ ve	Prevalence % (95% CI)	OR (95% CI)	χ2	df	*P*-value
Sex						12.6	1	0.3e-3 (< 0.01)*
Male	73	52	21	28.8(18.4–39.2)	Ref			
Female	353	171	182	51.6 (46.3–56.8)	2.6 (1.5–4.6)			
Breed						2.8	3	0.4
Ankole	69	40	29	42 (30.4–53.7)	Ref			
Crossed	77	44	33	42.9 (31.8–53.9)	1.1 (0.5–2)			
Sahiwal	23	13	10	43.5 (23.2–63.7)	1.1 (0.4–2.8)			
Frisian	257	126	131	50.9 (44.9–57.1)	1.4 (0.8–2.5)			
Age						19.7	2	5.1e-07 (< 0.01)*
Young	127	84	43	33.9 (25.6–42.1)	Ref			
Adult	210	88	122	58.1 (51.4–64.8)	2.7 (1.7–4.3)			
Old	89	51	38	42.7 (32.4–52.9)	1.5 (0.8–2.5)			
BCS						3.5	2	0.1
Poor	72	44	28	38.9 (27.63–50.2)	Ref			
Medium	127	60	67	52.8 (44.1–61.4)	1.8 (0.9–3.2)			
Good	227	119	108	47.6 (41.1–54.1)	1.4 (0.8–2.5)			

* − ve*, negative; + *ve*, positive; *95% CI*, confidence interval; *OR*, odds ratio; *χ2*, chi-square; *df*, degree of freedom; *, significant difference at *p*-value < 0.05, *BCS*, Body Condition Score

**Table 7:** Multivariate Logistic Regression Model Analysis of risk factors associated with bovine fasciolosis

Variables	ß-coefficients	*P*-value
Intercept	− 1.1841	< 0.01**
Sex	0.7430	0.01*
Age	0.5480	0.02*
Body condition score	0.4791	0.08
Breed	− 0.3579	0.21

With respect to cattle raising practices, the risk factors assessed were based on the feeding system, source of fodder, source of water and anthelmintics used to treat fasciolosis. Therefore, this study showed a high prevalence of fasciolosis among cattle drinking water from rivers or lake or irrigation canals (58.6%) than the cattle drinking water from the tap (40.9%). The univariate analysis showed that source of water for watering the cattle (χ2 = 12.7, *P*-value = 0.4e-03 means < 0.01) was statistically associated with the bovine prevalence of fasciolosis in this study. Furthermore, cattle drinking water from rivers or lake or irrigation canals (OR = 2.1; IC: 1.4–3.1) were likely to be more at risk to get bovine fasciolosis in the study area (Table 8).

**Table 8:** Univariate analysis and Estimated Odds Ratio (OR) for risks factors associated with bovine fasciolosis based on practices among farmers during cattle raising in Imbo region

Risks factors	Level	− ve	+ ve	Prevalence % (95% CI)	OR (95% CI)	χ2	*P*-value
Feeding system of cattle	Mix of stall feeding and Free grazing	15	12	44.4 (25.7–63.2)	Ref	0.1	0.7
	Stall feeding	208	191	47.9 (42.9–52.8)	1.2 (0.5–2.5)		
Source of fodder to feed the cattle	Agriculture land/fodder crop	210	191	47.6 (42.7–52.5)	Ref	< 0.1	0.9
	Aquatic environment	13	12	48 (28.4–67.6)	1 (0.5, 2.3)		
Source of water for watering the cattle	Tap water	156	108	40.9 (34.9–46.8)	Ref	12.7	0.4e-03 (< 0.01)*
	Rivers/lake/Irrigation canals	67	95	58.6 (51.1–66.2)	2.1 (1.4–3.1)		
Anthelmintics commonly used to treat cattle against fascioliasis	Albendazole/ Nitroxinil	143	119	45.4 (39.4–51.5)	Ref	1.4	0.2
	Oxyclozanide (Zanil)/ Nitroxinil	80	84	51.2 (43.6–58.9)	1.26 (0.9–1.9)		

* − ve*, negative cattle; + *ve*, positive cattle; *95%CI*, confidence interval; *OR*, odds ratio; *χ*^*2*^, Chi-square *: significant

## Discussion

The current study assessed the prevalence of *Fasciola* spp. in cattle using two different approaches and potential associated risk factors of bovine fasciolosis in the Imbo Region of Burundi. Results from fecal analysis of cattle from different farms in the region indicate that bovine fasciolosis is endemic with an infection prevalence of 47.7%. This result is similar to prevalence reports in other areas of Africa, such as Central, Southern and Western provinces of Zambia with 48% (Phiri et al. 2005), Iringa District of Tanzania with 42.1% (Keyyu et al. 2006), Mardan District in Pakistan with 42.8% (Khan et al. 2020), and in Western Ethiopia (Tulu and Gojam 2018) with 39.1%. On the other hand, a study conducted by Abebe et al. (2018) in Southwestern of Ethiopia showed a lower prevalence (21.4%) compared with the one found in the current study, while, a study in Kware State north-central of Nigeria showed a higher prevalence (74.9%) (Elelu et al. 2016). The results obtained from cattle slaughtered in abattoirs from the study region, indicate that bovine fasciolosis is endemic with a prevalence of 33.2%. These results are higher than the prevalence of 12.3% found in abattoirs during routine meat inspection by veterinary services (Report from General Directorate of livestock in Burundi 2021). The difference of this prevalence could be due to non-regularly recording of parasitic diseases cases during routine meat inspection by veterinary services in abattoirs. In a recent study, Minani et al. (2023) reported a 13.0% prevalence of *Fasciola* spp. in slaughtered ruminants at Muyinga (Burundi). The difference between the two prevalence could be due to magnitude of samples size used in these two studies. Despite the livestock system used in the two study areas, the farmers of the Imbo region who live and raise their livestock near the lake, rivers, and the marshes where they grow rice, have a habit of drawing water for watering livestock in these areas without being treated. The later result is also in the same line to the prevalence of 30.9% reported by Elshraway and Mahmoud (2017) in an abattoir of El-Kharga of Egypt, 30% reported by Kusumarini et al. (2020) in Malang District, East Java in Indonesia, and 39.1% reported by Tulu and Gojam (2018) in Western of Ethiopia using the same method in abattoir. Other studies in the same line with the current study were reported by Mequaninit (2021) with the prevalence of 35% in Kombolcha ELFORA abattoir located in the North Eastern part of Ethiopia, reported by Bernardo et al. (2011) with the prevalence of 24.9% in the Southern Espirito Santo and reported by Mungube et al. (2006) with the prevalence of 26% in semi-arid coastal part of Kenya. The study carried out by Abunna et al. (2010) showed that the prevalence (14.0%) in Saddo municipal abattoir, Southern part of Ethiopia was lower than the current study. While, the study conducted by Opio et al. (2021) at Lira Municipality abattoir in Northern of Uganda showed that the prevalence (65.7%) was higher than the current study. The overall prevalence of bovine fasciolosis observed in farms and in abattoirs was almost the same in the study area. This could be explained by the same origin of the selected cattle in abattoir which was shared with the cattle selected for fecal samples. Furthermore, they shared the same ecology, climatic, geographical location, and they received the same type of veterinary service (treatment and prevention). This study indicated in coprological examination that female and adult cattle were more exposed to bovine fasciolosis compared to male and young cattle respectively; which converge with studies reported by Khan et al. (2020) and Opio et al. (2021). The females are more exposed than males due to poor physiological and immune status and susceptibility between sex may exist for fasciolosis prevalence as reported by Phiri et al. (2005). The lower prevalence among young cattle (< 2 year) could be because the cattle on this age have not started the period of reproduction means that the physiological status still comfortable and the immune status is still good. The higher prevalence in adult cattle (2–4 years) could be because these cattle are in the period of reproduction and sometimes the immune status can be lower. Regarding the older cattle, the prevalence is lower because the cattle can be attributed to the high immunogenicity of the parasite, which aids in stimulation of acquired immunity in old animals as reported by Opio et al. (2021). In addition, the low prevalence in old cattle could be due to severe fibrosis of the liver which weakens the movement of immature flukes, calcification and stenosis of bile duct that provide unfavorable environment for adult parasites and finally result in their expulsion as reported by Khan et al. (2020). Regarding farmers practices during cattle raising, the present study showed that the source of water was statistically significant in association with bovine fasciolosis. This prevalence among the source of water could be due to the farmers who are used to drawing water for watering livestock in the areas including canal irrigation, edge of rivers or lake and stagnant water because many farmers live and raise the livestock near the lake or rivers or the marshes where they grow rice. Therefore, due to the zoonotic character of fasciolosis, the farmers of the Imbo region are at risk to fasciolosis due to some vegetables including eggplant, tomatoes and cabbage which are growing in these areas, and which can be eaten in raw state or farmers could use to drink untreated water from these areas during rice growth activities. In addition, children are mainly at risk because they could drink contaminated water while swimming in irrigation channels, rivers, and lake. To solve this challenge, the integrated measures control strategies based on selected anthelmintics drugs therapy against fasciolosis and against intermediate host of the parasite should be implemented to reduce the disease. The strategic of use of fasciolicides and other anthelmintic drugs should be implemented to prevent anthelmintics drugs resistance. Further studies on the epidemiology of the parasite using molecular characterization, on the ecology and biology of its intermediate host and on the evaluation of effectiveness of fasciolicides and other anthelmintics drugs used in Burundi should be carried out for better control magnitude of the problem. In addition, study on human fasciolosis in Burundi should be carried out earlier due to its zoonotic character and the environmental risk factors to human identified above.

## Data Availability

The datasets generated and/or analyzed during the current study are available from the corresponding author on reasonable request.
